# Supplementary material for: Are carbon emissions trading and green financial instruments synergistic? -Comprehensive quantitative research based on content analysis
Source: PLoS One. 2024 Mar 7;19(3):e0298601. doi: 10.1371/journal.pone.0298601 (PMC10919601; doi:10.1371/journal.pone.0298601)
Supplement: S1 Data — (ZIP) [file pone.0298601.s001.zip › Data/地级市Z_F工作报告.zip!/┴╔─■/╠·┴δ╩╨/└┤╫╘╣1⁄2╓┌║┼í╛┬φ┐╦╩2╛▌═°í┐_2016.docx]

各位代表：

　　现在，我代表市人民政府向大会报告工作，请予审议，并请市政协委员和列席同志提出意见。

　　一、2015年暨“十二五”工作回顾

　　刚刚过去的一年，我们在省委、省政府和市委的正确领导下，团结和带领全市各族人民，积极应对严峻复杂的经济形势，始终保持干事创业有激情，千方百计破难题，求真务实谋发展,各项工作取得了新成绩。

　　——对外开放取得新成果。坚持南北企业同时招、中央企业重点招、境外企业境内招、嫁接项目优先招、别人不招我们招，市县乡三级干部齐上阵，开展各类招商活动294次，引进新项目195个。中利光电新材料、奥拓福水刀、铁岭保税物流中心、中电国际垃圾焚烧发电等244个亿元以上项目开复工。中航资本重组新兴药业、中航宝胜收购浦东线缆、中集安瑞科收购哈深冷、中丝集团与西丰美麟合作等32个嫁接改造项目顺利实施。长飞光纤光缆、比科新能源电池、嘉冠石化、金正大二期等一批项目竣工投产。铁西老工业区、晓明镇独立工矿区搬迁改造项目获中央财政补助资金1亿元，铁岭四平通辽经济协作示范区建设上升为国家战略。核准对外直接投资企业6家，铁煤建工获得对外工程承包资质。

　　——工业发展迈出新步伐。《中国制造2025铁岭行动纲要》启动实施，确定智能制造支撑项目和示范企业78个。在建10亿元以上工业项目6个，实施重点技改项目102个，铁岭县、清河区光伏项目并网发电。新增产业集群骨干企业3户，5个研发检测平台、3个企业技术中心晋升省级。新建院士工作站3个，组建省级产业技术创新联盟和创新平台5个，签订产学研合作协议50个，申请发明专利175件，促进科技成果转化25项，开发新产品242个。中航新兴药业起草的2个产品标准载入《中国药典》，23家企业产品获得辽宁省名牌产品称号。高新技术企业发展到42户，实现产品产值111亿元。软环境建设全面加强，兜底服务工作体系更加完善，帮助企业化解各类难题近千件。

　　——农业农村经济健康发展。沈铁现代农业示范带规划编制完成。粮食再夺大丰收，总产量达到92.7亿斤。蔬菜总产量实现600万吨，榛林总面积提高到113.4万亩。新建改造标准化畜牧养殖场50个，全市畜牧业产值实现280亿元。农产品加工园区发展到11个，规模以上农业产业化龙头企业实现销售收入276.2亿元。市级以上农民合作社达到481家，经营200亩耕地以上的家庭农场281家，土地流转面积居全省首位。完成农田水利骨干工程240项，实施节水灌溉工程12.7万亩。农业综合机械化水平提高到84%。气象防灾减灾能力明显增强。农业标准化认证面积573万亩，“三品”认证176个。西丰柞蚕丝成为国家地理标志保护产品，铁岭平榛入选全国农业标准化20年成果展。支西工作实现重大突破，辉山乳业、茅台保健酒等一批重大项目加快建设，先后获评为国家粮改饲试点县、可持续发展实验区、生态保护与建设示范单位和省级生态县。

　　——服务业发展势头良好。星悦南岸、哥伦布广场、龙源温泉度假村、禧徕乐国际家居、欧蓓莎商城等综合体项目积极推进。开原中心商贸区晋升省级服务业集聚区，新增限额以上服务业企业39户。东北物流城实现销售收入29亿元，辽宁二手车市场交易车辆4.2万台，东北亚（铁岭）现代农业物流会展交易中心主体工程基本完工。组建铁岭绿源农副产品物流配送中心，270余种农副产品进入省内外大中城市超市，我市被确定为2015年省级肉类蔬菜流通可追溯体系建设项目试点城市。旅游业“五个一”工程取得明显成效，新开发农家乐示范村8个、温泉旅游项目2个、滑雪场2个。莲花湿地AAAA级景区、凡河新区水系省级水利风景区通过专家评审，冠名沈阳至武汉、大连至哈尔滨2列高铁列车，开通引客入铁专列12列，成功举办中国荷花节、湿地灯会、过大年、满族颁金节等系列节会活动，“快乐铁岭·莲花湿地”品牌进一步叫响。银泰活力城、红石郡等房地产项目加快建设，施工面积1045万平方米。凡河新区电子商务产业集聚区入驻企业110家，铁岭县东北高新技术信息产业园、铁岭电子商务大厦、银州区互联网+创业大厦运行良好，苏宁铁岭馆、邮农丰、中珍网等电商平台上线运营。

　　——城乡环境进一步改善。城市总体规划修编工作全面完成，城镇化率增幅居全省第一位。维修改造县级以上公路427.1公里，辽河生态路一期工程竣工，孤懿线朱尔山辽河大桥主体完工。中心城区新改建供暖管网37.2公里、供水管网14.1公里、燃气管网61.7公里，新增绿地58.8公顷。凡河新区兴隆大船整体投入运营，全球名品购物中心、恒基兆业商业街主体完工，各类经营业户发展到5026户，常住人口达到16.2万人。开原滨水新城基础设施日臻完善，依水茗郡、清湖郡等项目开工建设。昌图滨湖新城、辽北新城初具规模，南湖新区47栋回迁楼基本完工。西丰县启动弃管楼改造三年计划，一批景观绿化工程竣工。调兵山市四区两街加快建设，城南新区10.2万平方米绿化工程完工。清河区集中供热工程基本完成，大清河跨河大桥加快建设。铁岭县新台子等重点中心镇建设有序推进。实施宜居乡村建设项目45个，修建文化广场4.8万平方米，绿化村屯107个，建设美丽乡村示范村37个。

　　——生态建设扎实推进。节能减排示范工作取得阶段性成果，22个示范项目开工建设，到位中央奖励资金8亿元。二氧化硫等主要污染物排放量均呈下降趋势。在全省率先启动生态红线划定工作。辽河生态治理成效显著，莲花湿地公园被确定为国家林业局“十三五”重点投入项目。铁西干渠、新开河河道等8个项目列入国家水生态文明城市试点项目库，涉水国控重点企业排污许可证核发工作全部完成。大气污染防治实现联防联控，火电企业全部完成除尘和脱硫脱硝改造，水泥行业落后产能全部淘汰，区域高效一体化供热体系加快建立。完成植树造林28.8万亩，治理闭坑矿山23座。22个乡镇、209个村被评为市级以上生态乡村。退耕还林和清理小开荒工作取得新进展，畜禽养殖企业污染治理工作走在全省前列。

　　——发展保障能力不断增强。向上争取各类无偿资金130亿元，增长6.6%。成功发行地方政府债券置换债务69.3亿元。累计筛选入库PPP项目101个，投资规模286亿元。盛京银行、中天证券开业运营，新设浦发银行小微银行等3家网点，建行西丰支行获批筹建，永安保险落户铁岭，农信社改制全面启动，信贷、非信贷融资突破200亿元，昌图县被列为全国农村承包土地经营权抵押贷款试点县。金融广场服务功能进一步完善，合作机构达到89个，注册用户发展到335家。企业挂牌上市孵化基地启动运行，新兴药业、中旭石化成功登陆新三板，辽宁国美等20户企业在区域股权中心挂牌。土地管理工作不断强化，项目建设用地得到保障。出台了支持优秀人才创新创业若干意见。中德职业教育合作基地正式成立，辽宁工程职业学院荣获全国职业院校技能大赛团队一等奖。我市被确定为国家老工业基地产业转型技术技能人才双元改革试点市。

　　——各项改革深入推进。大力简政放权，取消、下放和调整行政职权250项，对保留的1449项行政职权权责清单在政府网站公示。市县乡村四级联动、全程代办的服务体系全面建成，在省内率先实现全覆盖。商事制度改革稳步推进，全市内资市场主体发展到16.1万户。按时完成市县两级政府机构改革任务。以县级公立医院改革为重点的医疗卫生体制改革有序推进。铁岭县、凡河新区、高新区、台湾工业园的管理体制、财政体制和债权债务关系得到理顺。部门预算编制、非税收入收缴等财税体制改革不断深化。新一轮国资国企改革稳步实施，北方铝业集团成功重组。全省首家市级农村产权交易市场投入运行，完成农村土地确权登记160万亩。集体林权主体改革任务全面完成。

　　——社会事业繁荣发展。新建、改扩建标准化幼儿园20所，完成义务教育学校标准化建设72所，改造薄弱学校42所，多媒体进教室比例达到86%。清河区通过义务教育发展基本均衡县（区）国家验收。铁岭县高中被确定为辽宁省特色普通高中实验学校，铁岭师专与韩国加耶大学开展专升本合作办学。成功举办两岸友谊之声音乐节，我市11名师生在全球正体汉字书法大赛上获奖。铁岭欢歌广场演出、送戏下乡等文化活动丰富多彩，二人转《铿锵玫瑰》荣获全国曲艺新人新作展演银奖，微电影《鞋垫奶奶》、《最佳投资》荣获第三届亚洲微电影艺术节5个奖项，103幅工笔画作品入选全国美展并获奖，中央电视台书画频道走进铁岭，我市被誉为东北书画第一市。城乡基本公共卫生服务工作基础不断夯实，爱国卫生运动扎实推进，昌图县成功创建国家卫生县城。铁岭市第八届运动会成功举办，我市运动员赵慧馨获得第31届奥运会射击项目入场券。国防动员和双拥共建工作扎实开展，文明城市、文明单位、文明乡村三大创建活动深入实施，孝道铁岭建设成效显著，党洪义同志荣获全国道德模范提名奖。

　　——社会保持和谐稳定。实施省市重点民生实事69件，其中市人大七届四次会议确定的17件实事全面落实。维修改造农村中心敬老院9家，50%的城市社区建成老年人日间照料室和活动场所，90岁、100岁高龄老人月生活补助分别提高到150元和600元。光荣街南段顺利通车，新三线二期主体完工，黑龙江路连接线开工建设。维修农村公路320公里，建设一事一议村内道路619.3公里。实施暖房子工程460万平方米，改造弃管楼608栋，沙子沟等集中连片棚户区改造工程开复工，物业管理覆盖率达到90.1%。大剧院、美术馆主体工程完工。广裕街亮化全面完成。北排系统管线和路面工程竣工，南排系统铺设管线2100米。新农合政府补助标准提高到人均380元，一站式报销服务启动实施。城乡最低生活保障标准分别提高7.1%和12.1%，农村五保集中和分散供养标准分别提高12%和8%。困难家庭高校毕业生就业援助工作得到落实。为246所学校安装摄像头1230个，实现了校园安全实时监控。新建农村饮水安全工程112处，解决了10.2万人饮水不安全问题。

　　就业创业政策得到落实，安置实名制就业3万人，城镇登记失业率控制在3.8%以内。企业退休人员养老金提高11%，城乡居民基础养老金标准提高到每月85元，在全省率先开展困难人员缴纳养老保险费贴息贷款工作。城镇居民医保政府补助标准提高到380元，省内异地就医即时结算业务开通。实施残疾人家庭无障碍改造1422户，发放辅助器具7261件，志愿助残工作经验在全国推广。新建、改扩建200平方米以上村部338个、400平方米以上农村社区36个。7.9万农村贫困人口实现稳定脱贫。应急管理工作全面加强，安全生产网格化、信息化监管模式初步确立，食品药品安全形势稳定向好。市县乡三级干部全员接访工作机制、社会矛盾纠纷大调解体系更加完善，社会治安大局持续稳定，群众安全感连续4年保持全省第一。单户城镇化、金峰小镇、新老城区房照办理等一批遗留问题逐步得到解决。12345市长热线软件系统成为全省样板，民心网诉求办理工作居全省首位。政务公开、政务督查和绩效管理工作进一步加强。依法行政水平明显提升，政府廉政建设不断深化，“三严三实”专题教育取得明显成效。办理人大议案、人大代表建议和政协提案516件，见面率、办复率和满意率均达99.8%以上，落实率稳步提升。

　　民族、宗教、综合执法、审计、外事、侨务、人防、物价、地震、国家安全、国税、地税、武警、消防、邮政、通信、烟草、检验检疫、供电、档案、驻外联络、市志、老龄、仲裁等工作都取得了新成绩。

　　随着2015年的过去，我们走过了不平凡的“十二五”。五年来，我们在困境中奋进，在拼搏中前行，谱写了幸福美好新铁岭建设的新篇章。国民经济逐步走上健康发展轨道，按照“三严三实”要求，全面做实各项数据，预计地区生产总值实现745亿元，一般公共预算收入50.3亿元，固定资产投资396亿元，规模以上工业增加值118亿元，社会消费品零售总额407亿元，真实反映了经济发展水平，为未来发展奠定了坚实基础。对外开放开创新局面，招商引资上项目成为全市共识，累计引进域外投资项目近千个，70个国内外500强企业落户铁岭，25户企业纳税额超千万元，8个省级工业产业集群和8个省级服务业集聚区规模进一步做大，农业产业化水平不断提高，经济运行质量持续提升。城乡面貌焕然一新，中心城区功能日臻完善，老城区改造持续推进，凡河新区成为铁岭的靓丽名片，县城规模不断扩大，重点中心镇建设步伐加快，四横五纵六通路网格局基本形成，城乡人居环境明显改观，绿色发展方式和生活方式深入人心。社会事业全面繁荣，各级各类教育均衡发展，连续3届获得全国教育改革创新特别奖，被确定为中国教育优先发展示范城市，城乡医疗卫生服务体系基本建成，文化体育事业蓬勃发展，孝道文化建设走在全国前列，公民文明素质不断提升。民生福祉持续改善，累计完成民生实事185件，城乡居民人均可支配收入分别达到20625元和11655元，就业形势总体平稳，统筹城乡的社会保障体系基本建立、保障标准逐年提升，人民群众的幸福感和获得感显著增强。

　　五年来，铁岭发展取得来之不易的成绩，主要得益于我们始终保持发展定力，一张蓝图绘到底，一个思路贯始终，持之以恒抓招商，千方百计上项目，形成了实事求是、真抓实干的工作作风，走出了一条符合铁岭实际的科学发展之路；得益于我们始终坚持向改革要红利、要活力、要动力，牢牢把握发展新常态，努力突破体制机制束缚，树立了勇于实践、善于创新的工作思维，为新一轮振兴发展积蓄了力量；得益于我们牢固树立均衡发展、绿色发展理念，始终坚持统筹兼顾，积极破除城乡二元矛盾和资源环境约束，促进区域协调发展、人与自然和谐共生，实现了更高质量、更加公平、更可持续的发展；得益于我们始终坚持民生优先，确立了以人为本、共建共享的工作目标，全力实现好、维护好、发展好最广大人民的根本利益，不断加强社会建设，努力维护公平正义，调动了全市人民的积极性、主动性和创造性；更得益于我们始终坚持市委的正确领导，自觉接受市人大依法监督、市政协民主监督，与全市人民一道谋发展、促振兴，营造了政通人和、干事创业的浓厚氛围，汇聚了幸福美好新铁岭建设的强大合力。在此，我代表市人民政府，向辛勤工作在各条战线上的全市各族人民，向各民主党派、各人民团体和各界人士，向解放军驻铁部队指战员和武警官兵，向所有关心、支持、参与幸福美好新铁岭建设的社会各界朋友，表示衷心的感谢并致以崇高的敬意！

　　在看到成绩的同时，我们也清醒地认识到全市发展中存在的矛盾和问题。

　　一是缺少可以支撑全市发展的大项目和项目群，经济发展的后劲有待增强。

　　二是结构调整任务依然艰巨，大农业、小工业、小服务业的格局没有发生根本改变。

　　三是摆脱财政困难局面任重道远，保刚性、保民生、保运转、保偿债的压力持续增大。

　　四是政府自身建设有待加强，群众利益诉求增多，社会维稳任务依然繁重。这些问题已经引起我们高度重视，并将在今后的工作中认真加以解决。

　　二、“十三五”指导思想和发展目标

　　“十三五”时期是全面建成小康社会的决胜阶段，也是全面深化改革、加速转型升级的关键时期。总的指导思想是：深入贯彻习近平总书记系列重要讲话精神，落实“四个全面”战略思想，牢固树立创新、协调、绿色、开放、共享发展理念，坚持以“四个着力”为根本遵循，紧紧依靠“四个驱动”共同发力，培育壮大“六个新的增长点”，以对外开放招商引资上项目为抓手，加快推进新型工业化、新型城镇化、信息化和农业现代化，繁荣文化，保障民生，加强法治，走出一条质量更高、效益更好、结构更优、优势充分释放的发展新路，到2020年全面建成小康社会，实现美丽富庶文明快乐新铁岭奋斗目标。

　　主要发展目标是：

　　——综合经济实力全面提升。人均地区生产总值比2010年翻一番，年均增长7%。一般公共预算收入年均增长7%以上；固定资产投资年均增长10%；社会消费品零售总额年均增长9%。经济结构优化升级，服务业增加值占地区生产总值的35%以上, 先进制造业增加值占工业增加值的60%以上，农业现代化水平步入全国先进行列。城乡结构趋向合理，常住人口城镇化率达到55%以上。

　　——发展动力与活力明显增强。非公有制经济增加值占地区生产总值的80%以上，高新技术产值年均增长30%，研究和发展经费占地区生产总值的1.5%，科技进步贡献率达到50%以上，每万人发明专利拥有量达到1.8件，互联网普及率达到100%。

　　——生态环境质量持续提升。单位生产总值能耗下降15%，单位生产总值二氧化碳降低率达到3%，耕地保有量54.3万公顷，森林覆盖率达到35.38%，中心城区建成区人均公共绿地面积达到13平方米。

　　——人民生活水平普遍提高。城乡居民收入年均增长7%。新增劳动力受教育年限达到12年，城镇新增就业5万人，城乡社会保障全覆盖，贫困地区人口全面脱贫。

　　做好“十三五”时期工作，必须做到以下“六个坚持”：

　　一是坚持开放发展。抢抓“一带一路”、新一轮东北振兴、沈阳经济区等战略叠加机遇，积极融入国家发展战略，推动跨省经济合作。坚持招商引资上项目不动摇，推进引资、引技、引智有机结合，促进外向型经济发展，实现对外开放战略性和实效性的新突破，打造内陆开放城市。

　　二是坚持创新发展。加强理论创新，解放思想，更新理念，破解经济社会发展的思想瓶颈。实施供给侧结构性改革，推进制度创新，破除体制机制弊端，充分发挥市场在资源配置中的决定性作用，更好发挥政府作用，逐步实现治理体系和治理能力现代化。强化科技创新，突出企业创新主体地位，激发大众创业、万众创新活力，促进新技术、新产业、新业态蓬勃发展，形成推动发展新动力，建设创新型城市。

　　三是坚持转型发展。切实转变经济发展方式，妥善处理好速度与结构、质量、效益的重大关系。以信息化为引领，统筹推进新型工业化、城镇化、农业现代化发展，加快构建以品牌农业、智能制造业、现代服务业为支撑的现代产业体系。扩大消费投资，推动消费结构升级，实现投资由规模速度型增长向质量改善型转变，打造铁岭经济升级版。

　　四是坚持协调发展。统筹区域协同发展，推进城乡一体化，实现新老城区双城并盛，促进物质文明和精神文明并重、经济建设和社会建设融合，在协调发展中补齐短板、拓宽空间，走出具有铁岭特色的统筹推进、融合发展新路子。

　　五是坚持绿色发展。大力发展绿色经济、低碳经济和循环经济，强化生态环境治理、资源综合利用，推进节能减排，形成节约资源和保护环境的空间格局、产业结构和生产生活方式，实现人与自然和谐共生、经济社会可持续发展，努力建设山青、水秀、天蓝、地绿的美丽家园。

　　六是坚持共享发展。把增进人民福祉作为全面建成小康社会的出发点和落脚点，繁荣发展各项社会事业，弘扬以快乐为特征的地域文化，兜底基本民生，畅通民意诉求渠道，建立稳定的公共财政投入保障机制，完善公共服务体系，推进城乡公共服务均等化，维护社会公平正义。持续增加城乡居民收入，实施精准扶贫脱贫，让全市人民更广泛地参与发展过程、更公平地分享发展成果。

　　三、2016年工作安排

　　主要预期目标是：地区生产总值增长5%，一般公共预算收入增长2%，固定资产投资增长6.5%，社会消费品零售总额增长8%，城乡居民人均可支配收入分别增长7%。重点抓好以下10项工作：

　　（一）深入开展项目年活动，全方位扩大开放

　　坚持项目牵动战略，以有效投资拉动经济的快速发展。继续实施“五招”，深耕吉黑、京津冀、长三角、珠三角地区，着力抓好保定造纸企业和通州产业转移项目，开展大型招商活动20次以上，力争引进5000万元以上项目100个以上，到位内资增长8%以上。铁岭保税物流中心封关运行，铁路物流基地、铁西老工业区和晓明镇独立工矿区搬迁改造项目加快建设，争取大明镇独立工矿区进入国家试点、100万吨玉米乙醇项目开工建设。扎实推进铁岭四平通辽经济协作示范区建设，主动融入“一带一路”倡议。进一步巩固和加强软环境建设，落实领导包项目责任制，全力抓好项目开复工，力争茅台保健酒、中利光电新材料、中电国际垃圾焚烧发电、哥伦布广场等100个亿元以上项目竣工投产。实施对台重大项目攻坚计划，各县（市）区至少引进建设1个台资项目。大力发展外向型经济，推动汽车零部件、农畜产品、外贸综合服务和鹿产品出口基地建设，支持清河石油机械、调兵山鑫丰矿电、铁岭县圣加仑等优势企业“走出去”。

　　（二）实施制造强市战略，推动工业转型升级

　　深入落实《中国制造2025铁岭行动纲要》，在数字车间、智能产品等6大领域实施智能制造示范工程，争创省级两化融合贯标示范企业2户。促进大数据与工业互联网、个性化定制等融合集成，推动制造模式变革，培育新业态。积极引进世界500强和高技术企业，大力发展光伏发电、新能源汽车等战略性新兴产业。积极争取国家和省股权投资基金投入，重点支持发展潜力大的现有企业，实施2000万元以上技改项目50个，新增规模以上工业企业50户。积极稳妥化解过剩产能，嫁接重组僵尸企业和停建项目40个，工业产能利用率提升5%。推动产业集群纵向延伸产业链条、横向组建产业联盟，每个产业集群至少新增1户骨干企业。继续开展质量年活动，进一步加强标准化建设。发挥创新引领作用，实施产学研合作攻关工程，推动8户企业产品换代升级，新建院士工作站1家、科技公共服务平台2个，创建产业技术创新联盟2个，组建创新创业导师团队20个。申请发明专利200件，转化科技成果35项，开发新产品350个，努力建设专利使用大市。新增高新技术企业5户，全市高新技术产品产值实现122亿元。深入实施兜底服务，全面落实支持企业发展各项政策措施，开展降低企业成本专项行动，进一步加强软环境建设，助推企业发展。

　　（三）提高农业现代化水平，打造铁岭农业品牌

　　坚持用工业理念谋划农业，着力抓好“五个一”工程，推动铁岭由农业大市向农产品精深加工大市、食品工业大市转变。全面启动沈铁现代农业示范带建设，重点实施九大工程33个重点项目，抓好水稻、玉米、蔬菜、榛子、梅花鹿等精品示范区，科学调整烟草、花生、胡萝卜等种植规模，每个县（市）区建设一个规模较大的认领农业园区和一个农产品电子商务中心，打造全国知名的绿色农产品生产基地，争做沈阳等大城市的粮仓、菜篮子和后花园。加强农田水利基本建设，提高农业机械化水平和气象服务能力，全力夺取粮食大丰收。加快推进规模养殖企业升级改造，打造全省清真食品加工基地。加快培育新型农业经营主体，创新土地流转形式，促进规模适度经营，新增市级以上农民合作社50家、家庭农场100家，土地流转面积达到400万亩。每个县市重点建设一个农产品加工园区，重点推进辉山乳业、昌图农高区、铁岭县农科园、九三物流园等项目建设，规模以上农业产业化龙头企业发展到130家以上，其中农产品加工企业110家以上，销售收入增长5%以上。建立和完善农业信息网络。完成“三品”认证180个，农业标准化认证面积570万亩以上。加大农村环境治理力度，逐步推进垃圾无害化处理，提高村屯绿化水平，每个县（市）区至少抓一个在全省领先的高标准宜居乡村示范典型。

　　（四）大力发展现代服务业，释放消费潜力

　　坚持现代服务业发展与城镇化建设、产业发展和创新创业相结合，通过引进项目特别是大型载体实现产业扩张和升级，加快培育新的发展动能，提高消费水平。进一步做大服务业集聚区规模，推动禧徕乐国际家居、上海赢信财富广场、辽北居然城、恒基兆业商业街等综合体建设，新增限额以上服务业企业35户以上。充分发挥保税物流、铁路物流、邮政物流、检测追溯和供销系统组织作用，抓好东北物流城运行，支持新天公司第三方支付平台发展，加快推动东北亚（铁岭）现代农业物流会展交易中心建设，继续加大农超对接力度，建设特色淘宝村，构建集线上线下运营于一体的现代农业物流体系，努力形成农产品区域集散中心和保障基地。继续推进旅游业“五个一”工程，加快落实一县一个温泉度假村、一个大的旅游景点、一个特色农家乐、一个旅游纪念品，有条件的建设一个滑雪场，办好系列旅游节会活动，打造铁岭特色旅游品牌。抓好莲花湿地三期、星悦南岸水世界、万亩花海核心景观区等项目建设，推动凡河新区水系创建国家级水利风景区。加强与国旅等大型旅行社合作，推进互联网+旅游平台建设，整合旅游资源，规划精品线路，开通引客入铁专列30列以上，形成特色生态游、文化游、乡村游产业链，构建大旅游发展新格局。推动工业企业主辅分立，着力发展生产性服务业，积极引导家政、养老、健康、文体等社区商业发展。

　　（五）推动城乡协调发展，提高新型城镇化水平

　　按照宜居宜业、方便生活、城乡繁荣的目标，统筹抓好规划、建设、管理三大环节，促进有能力在城镇稳定就业和生活的农业转移人口进城落户。积极推进沈铁城际铁路和102国道改线工程建设，新三线二期全面竣工通车。强化城市规划的指导性，协调推进智慧城市和数字城市建设，不断完善中心城区街道、社区服务体系，规范物业管理机制，努力提高精细化管理、规范化服务水平。积极化解房地产库存，推动有意愿的开发商启动建设，加大棚户区改造货币化安置力度，房地产施工面积770万平方米。进一步完善凡河新区功能，加快构建社会管理体系和产业支撑体系，促进各类资源合理配置，打造产城融合的生态宜居城市。提升开原滨水新城水系和景观效果，增强吸纳人口能力。抓好昌图滨湖新城金月湾大道、政府路西拓等路网工程，完成实验学校等项目建设。推进西丰县寇河湿地公园二期、三期工程，提高生态水平。完成清河区集中供热改造工程，加快建设大清河文化旅游产业综合发展带。加快调兵山市城南新区和铁岭县新台子基础设施建设，提升综合承载能力。统筹推进八面城、庆云堡等重点中心镇建设，打造优良人居环境，吸引周边农村居民入住。

　　（六）积极保护资源环境，建设美丽铁岭

　　继续抓好国家节能减排示范市、水生态文明试点市建设，深入实施四大生态工程，确保单位生产总值能耗和主要污染物排放量持续下降。抓好大气污染联防联控，取缔城市建成区10吨以下燃煤锅炉，推进铁岭电厂、清河电厂供热改造和一县一热源建设，加大秸秆转化利用、施工扬尘治理、黄标车整治力度，提高优良天气水平。启动水污染防治行动计划，对县级以上污染源进行许可证管理，完成调兵山市城南污水厂和西丰县两座污水厂的升级改造。加大畜禽养殖粪污治理力度，建设全省生态畜牧业示范市。加强辽河保护区生态治理工程建设，完成13条支流河口污染防治任务，促进辽河水质明显改善。实施三北防护林和退耕还林工程，植树造林18.3万亩，逐步改善矿区、生态脆弱区和敏感区生态环境。

　　（七）加强财税金融工作，提高发展保障能力

　　落实营改增、小微企业税收减免等各项财税政策，加强对重点税源企业和重点行业的动态监控，全面清理欠税，确保应收尽收。遵循先有预算、后有支出的原则，做好预决算和三公经费公开，严格执行人大批准的支出预算，严禁超预算或无预算支出。建立健全地方政府性债务管理体系、责任体系和风险防控体系，推行政府债务管理预算化，争取更多地方政府债券发行额度，全力化解存量债务。进一步扩大金融广场服务内容和范围，新增合作机构3个以上，新发展注册用户80家以上。依托企业挂牌上市孵化基地，助推3-5户企业实现挂牌。大力推进农信社改制工作，逐步完善金融服务体系。加大向上争取资金力度，到位各类无偿资金100亿元以上。强化节约集约用地动态监管，加强全过程执法监察，提升土地综合利用水平。做好海外研发团队引进、高技能人才培训工作，加强技能大师工作室建设，为经济社会发展提供人才智力保障。

　　（八）深化改革创新，增强发展动力和活力

　　加大简政放权力度，再取消和下放一批行政职权，建立健全权责清单动态调整机制。抓好政务服务载体建设，优化建设项目审批流程，提升政务服务质量和效能。稳步推进国库集中支付、国税地税征管、公务卡管理制度、政府购买服务和公务用车改革。大力推进PPP模式，建立健全项目库，吸引社会资本参与城市公共服务领域和基础设施建设。继续推进商事制度改革，适时启动工商登记网上审批、电子营业执照发放和企业简易注销程序，积极引导大众创业、万众创新，新发展私营企业1000户、个体工商户5000户以上。对市属经营性国有资产进行集中统一监管，实施政企脱钩和混合所有制企业改革试点。启动供销合作社综合改革工作。深化集体林权制度改革和国有林场改革，推进林权抵押贷款和森林保险工作。建立健全市县乡三级农村产权交易市场，完成土地确权登记颁证面积200万亩。持续推进保护性耕作制度改革，推广翻转犁深耕填埋、秸秆还田深松等技术模式。继续抓好县级公立医院改革，打好深化医改惠民攻坚战。稳步推进工资收入分配制度改革，落实劳动用工备案制度，建设和谐劳动关系。

　　（九）增进民生福祉，繁荣社会事业

　　按照坚守底线、突出重点的原则，多渠道筹措资金，为全市人民办好26件实事。1.继续实施孝道铁岭建设。2.打好脱贫攻坚战。3.提高最低工资标准。4.提高城乡最低生活保障标准。5.提高新农合政府补助标准，全面实施新农合和大病保险“一站式”报销服务。6.为困难人员缴纳养老保险办理贴息贷款。7.扶持创业就业，确保零就业家庭动态为零。8.建设教育资源公共服务平台。9.实施文化惠民工程。10.建设肉菜流通追溯体系试点。11.实施机动车尾气排放达标工程。12.建设美丽乡村示范村。13.维修改造农村道路500公里。14.改造弃管楼329栋。15.改造棚户区30万平方米。16.实施城市供水2025计划。17.改造城市燃气管网。18.建设垃圾焚烧发电项目。19.实施中心城区河湖连通生态治理。20.实施中心城区公共交通畅通工程。21.继续建设黑龙江路连接102线道路。22.改造光荣街。23.改造东北城大道。24.扩建高铁铁岭西站广场。25.建设环路城南段。26.维修改造银州区小街小巷5万平方米。

　　继续开展教育年活动，建设连锁幼儿园100所，改善贫困地区义务教育薄弱学校基本办学条件，加快发展现代职业教育，争取市直和银州区捆绑创建全国义务教育发展基本均衡县（市）区通过国家验收。广泛开展志愿服务活动，抓好乡村学校少年宫建设。继续开展铁岭欢歌、送戏下乡、秧歌剧推广等文化活动，办好诗词和文学作品大赛，推动书画和微电影创作，打造东北书画第一市和小品小戏特色市。开展全民健身活动，启动全省第九届少数民族传统体育运动会筹备工作。继续办好满族颁金节，大力开发嘎拉哈等世居民族文化产品。落实全面二孩政策，加大农村改厕力度，实施农村社区卫生服务提升工程，确保通过省级卫生城复核验收。进一步加强城市社区网格化管理和社会化服务工作。抓好残疾人助行和家庭无障碍改造工程。加强信访工作，完善12345市长热线系统运行和民心网诉求办理机制，妥善解决涉及群众切身利益的实际问题。加大应急管理力度，落实安全生产政府监管责任和企业主体责任，强化食品药品安全监管。扎实推进平安建设四级联创，进一步完善立体化社会治安防控体系,实现公共安全管理精细化、信息化、法治化。加强国防建设和国防教育，合力推进军民融合发展。

　　（十）加强政府自身建设，提升执政能力和水平

　　各级政府及其工作人员必须自觉做到尊法、学法、守法、用法，把政府工作全面纳入法治轨道。自觉接受人大及其常委会的监督，认真落实人大及其常委会的各项决议决定，坚持向人大及其常委会报告工作。主动加强同人民政协的联系，积极支持人民政协履行政治协商、民主监督和参政议政职能。认真办理人大议案、人大代表建议和政协提案。注重听取民主党派、工商联、无党派人士等方面的意见和建议，积极推进决策科学化、民主化，让所有工作接受人民监督、体现人民意愿。严格落实党风廉政建设主体责任和监督责任，坚决遵守《条例》和《准则》，加强行政监察，发挥审计监督作用，扎住制度围栏，打掉寻租空间，铲除腐败土壤。认真践行“三严三实”要求，大力弘扬实事求是、求真务实的优良作风，全面推行政务公开，进一步强化政务督查，加快完善绩效评价体系，一切从铁岭实际出发，凡事说了就算、定了就办、办就办好，确保政令畅通、令行禁止，以踏踏实实的作风、实实在在的业绩取信于民。牢固树立以人为本的执政理念，始终把为人民谋发展增福祉作为最大责任，始终把美丽富庶文明快乐新铁岭建设使命扛在肩上，始终把群众冷暖安危忧乐放在心头，切实增强服务意识，努力改进管理方式，主动为企业、为群众、为社会做好服务，不断开创政府工作的新局面。

　　各位代表，我们已经踏上了“十三五”的新征程，前景光明，任务繁重。让我们更加紧密地团结在以习近平同志为核心的党中央周围，在中共铁岭市委的正确领导下，万众一心，砥砺前行，奋力推进美丽富庶文明快乐新铁岭建设，为全面建成小康社会而努力奋斗！
